# One-year outcomes following operative or non-operative management of adhesional small bowel obstruction

**DOI:** 10.1093/bjsopen/zrad103

**Published:** 2023-10-14

**Authors:** Marie R Mortensen, Mohammad Alouda, Zara Bond, Jakob Burcharth, Katrine F Finne, Thomas K Jensen, Ida Lolle, Talha Malik, Loan Ngo-Stuyt, Liv B J Nielsen, Maria Olausson, Anders P Skovsen, Mette A Tolver, Henry G Smith

**Affiliations:** Digestive Disease Center, Bispebjerg and Frederiksberg Hospital, University of Copenhagen, Copenhagen, Denmark; Department of Gastrointestinal and Hepatic Diseases, Surgical Division, Herlev and Gentofte Hospital, University of Copenhagen, Copenhagen, Denmark; Department of Surgery, Hvidovre Hospital, University of Copenhagen, Copenhagen, Denmark; Department of Gastrointestinal and Hepatic Diseases, Surgical Division, Herlev and Gentofte Hospital, University of Copenhagen, Copenhagen, Denmark; Department of Surgery, Nordsjællands Hospital, University of Copenhagen, Hillerød, Denmark; Department of Gastrointestinal and Hepatic Diseases, Surgical Division, Herlev and Gentofte Hospital, University of Copenhagen, Copenhagen, Denmark; Department of Surgery, Hvidovre Hospital, University of Copenhagen, Copenhagen, Denmark; Department of Surgery, Slagelse Hospital, Slagelse, Denmark; Department of Surgery, Sjælland University Hospital, Køge, Denmark; Digestive Disease Center, Bispebjerg and Frederiksberg Hospital, University of Copenhagen, Copenhagen, Denmark; Department of Surgery, Sjælland University Hospital, Køge, Denmark; Department of Surgery, Nordsjællands Hospital, University of Copenhagen, Hillerød, Denmark; Department of Surgery, Sjælland University Hospital, Køge, Denmark; Digestive Disease Center, Bispebjerg and Frederiksberg Hospital, University of Copenhagen, Copenhagen, Denmark; Department of Surgery, Slagelse Hospital, Slagelse, Denmark

## Abstract

**Background:**

A trial of initial non-operative management is recommended in stable patients with adhesional small bowel obstruction. However, recent retrospective studies have suggested that early operative management may be of benefit in reducing subsequent recurrences. This study aimed to compare recurrence rates and survival in patients with adhesional small bowel obstruction treated operatively or non-operatively.

**Methods:**

This was a prospective cohort study conducted at six acute hospitals in Denmark, including consecutive patients admitted with adhesional small bowel obstruction over a 4-month interval. Patients were stratified into two groups according to their treatment (operative *versus* non-operative) and followed up for 1 year after their index admission. Primary outcomes were recurrence of small bowel obstruction and overall survival within 1 year of index admission.

**Results:**

A total of 201 patients were included, 118 (58.7 per cent) of whom were treated operatively during their index admission. Patients undergoing operative treatment had significantly better 1-year recurrence-free survival compared with patients managed non-operatively (operative 92.5 per cent *versus* non-operative 66.6 per cent, *P* <0.001). However, when the length of index admission was taken into account, patients treated non-operatively spent significantly less time admitted to hospital in the first year (median 3 days non-operative *versus* 6 days operative, *P* <0.001). On multivariable analysis, operative treatment was associated with decreased risks of recurrence (HR 0.22 (95 per cent c.i. 0.10–0.48), *P* <0.001) but an increased all-cause mortality rate (HR 2.48 (95 per cent c.i. 1.13–5.46), *P* = 0.024).

**Conclusion:**

Operative treatment of adhesional small bowel obstruction is associated with reduced risks of recurrence but increased risk of death in the first year after admission.

**Registration number:**

NCT04750811 (http://www.clinicaltrials.gov).prior (registration date: 11 February 2021).

## Teaser

Most patients with adhesional small bowel obstruction are treated non-operatively. However, recent registry studies suggest that operative management may be of benefit, reducing the risk of recurrent small bowel obstruction. In this prospective multicentre study, we found that operative treatment of adhesional small bowel obstruction was associated with reduced risk of recurrence but increased risk of all-cause mortality rate.

## Introduction

Small bowel obstruction (SBO) is a common general surgical emergency, thought to be responsible for up to 20 per cent of all acute surgical admissions^1^. Intra-abdominal adhesions are by far the most common cause, with adhesional SBO (aSBO) accounting for roughly 60 per cent of all cases^2,3^. Although they may also occur in patients with so-called ‘virgin abdomens’^4^, previous surgery is the most common cause of adhesions, which occur in over 30 per cent of patients undergoing surgery, with greater risks associated with an open rather than a laparoscopic approach^5–7^. Given this association, the surgeon has often been thought of as the pathogen in the development of aSBO, with surgery to be avoided if at all possible. In recent times, non-operative management has been the internationally favoured approach in stable patients with aSBO, with urgent operations reserved for those with suspected closed loop obstructions, bowel ischaemia or perforation^8–10^.

The results of recent studies suggest that early operative intervention may actually be of benefit for patients with aSBO, casting doubt on these previous assumptions. A large retrospective registry study from Canada found that operative intervention was associated with a lower overall risk of recurrent SBO when compared with non-operative management (13.0 per cent *versus* 21.3 per cent, *P* <0.001)^11^. A similar reduction in the risk of recurrence (from 19.0 per cent *versus* 25.6 per cent, *P* <0.005) was found in another registry-based study from America^10^. These data suggest that the benefit of dividing symptomatic adhesions that have caused obstruction may outweigh the risks of new adhesion formation.

Retrospective studies comprise the majority of previous research regarding recurrence of aSBO^9–12^. In addition to the risks of misclassification bias, these studies are limited by a lack of clinical data, particularly regarding the patient’s past surgical history. The Danish Audit of Small Bowel Obstruction (DASBO) study is a multicentre prospective study that included consecutive patients admitted with SBO of any cause. Here, the 1-year outcomes of patients included in the DASBO study with aSBO are reported, with the aim of comparing the impact of initial treatment strategy on recurrence rates and long-term mortality rate.

## Methods

The DASBO study was a multicentre prospective cohort study that included consecutive patients admitted to six acute hospitals in Zealand, the most populous island in Denmark with approximately 2.3 million inhabitants. Patients aged ≥18 years with a radiological or clinical diagnosis of SBO were eligible for inclusion. The study was registered on clinicaltrials.gov (NCT04750811), approved by the Danish Data Protection Agency (P-2021-70), and consent was obtained from all participating patients. Reporting was conducted according to STROBE guidelines^13^.

DASBO included patients with SBO of any cause, whose short-term outcomes have already been reported^3^. The present study only included those patients admitted with aSBO. Clinicopathological data were retrieved from electronic patient records and entered in a pseudoanonymized format into a secure REDCap database housed by the Capital Region of Denmark, which was only accessible to the study team. The original admission with aSBO during the DASBO study interval is referred to as the index admission, regardless of whether patients had previous episodes of aSBO. Patients were stratified according to their management during that index admission (operative *versus* non-operative) and according to whether they had previous episodes of aSBO (primary *versus* recurrent).

Follow-up was conducted using electronic patient records following patient discharge. All hospitals in Zealand use the same system for electronic patient records, allowing readmissions in any of the hospitals in this region to be identified. Furthermore, electronic patient records in Denmark are automatically updated in the case of a patient’s death, allowing mortality rates to be calculated with certainty. Data was checked for completeness by the principal investigator and validated by the local investigators for each centre.

### Outcomes of interest

The primary study endpoints were recurrence of SBO and overall survival within 1 year of index admission. Recurrences were defined as a readmission with a clinical or radiological diagnosis of SBO. Secondary endpoints included the number of SBO recurrences and the treatment of each recurrence.

### Statistics

Descriptive statistics comparing clinicopathological demographics between groups were performed using the chi-square test for categorical data and the Mann–Whitney *U* test for continuous data. Overall survival was calculated using the Kaplan–Meier method and compared using the log-rank test. To identify prognostic factors for SBO recurrence and all-cause mortality rate, univariable and multivariable Cox regression analyses were performed. The following factors were investigated for both outcomes and were chosen a priori: age; sex; ASA grade; performance status; Charlson Co-morbidity Index (CCI); treatment type (operative *versus* non-operative); number of previous operations and previous episodes of aSBO. Suspected bowel ischaemia on computed tomography (CT) scanning was included as an additional factor for all-cause mortality rate. The results of the Cox regression analyses are presented as hazard ratios (HR) with 95 per cent c.i. All analyses were performed using SPSS version 25.0 (IBM, Armonk, New York, USA).

## Results

**Fig. 1:**
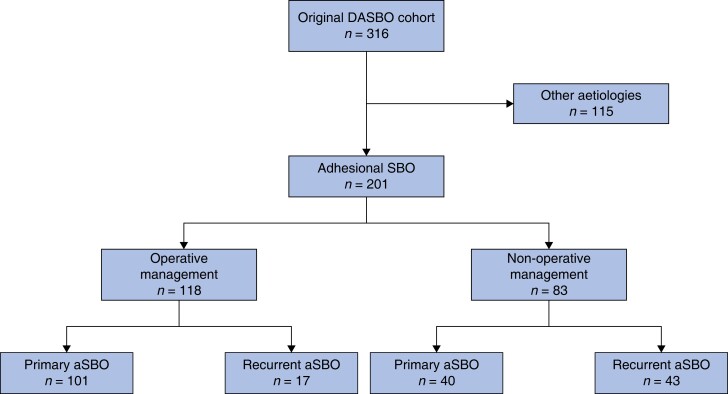
Consort diagram of the study cohort DASBO, Danish Audit of Small Bowel Obstruction; aSBO, adhesional small bowel obstruction.

A total of 201 patients were included in the present study, 141 (70.1 per cent) of whom were admitted with their first ever episode of aSBO (*Fig. 1*). No patients were lost to follow-up. All but one patient was diagnosed using abdominal CT scanning. The majority of patients underwent operative management during their index admission (118 patients, 58.7 per cent), of whom 17 underwent delayed surgery after an initial trial of non-operative treatment. Clinicopathological demographics of patients according to the management of their index admission is shown in *Table 1*. No difference in ASA grade or CCI was noted between groups, although patients managed non-operatively had poorer performance status. These patients were also more likely to have had previous admissions with aSBO. The clinicopathological demographics of the patient cohort, stratified into those with primary or recurrent aSBO, are summarized in *Table 2*. Whilst no differences in age or co-morbidities were noted between these groups, the number of previous operations was significantly higher in patients with recurrent aSBO.

**Table 1:** Clinicopathological demographics of patients treated operatively or non-operatively on index admission for adhesional small bowel obstruction

	Treatment of aSBO		*P*
	Operative	Non-operative	
No. of patients	118	83	–
Male:female	48:70	37:46	0.582
Median age (i.q.r.)	73 (57–82)	64 (55–78)	0.064
**ASA grade**			
I	22 (18.6)	9 (10.8)	0.159
II	52 (44.1)	38 (45.8)	–
≥III	37 (31.3)	36 (43.4)	–
**Performance status**			
0	63 (53.3)	40 (33.9)	**0**.**016**
1	38 (32.2)	16 (19.3)	–
≥2	19 (16.1)	26 (31.3)	–
Median CCI (i.q.r.)	3.5 (2.0–5.0)	2.0 (4.0–6.0)	0.352
Median previous operations (i.q.r.)	1 (0–2)	2 (1–4)	**<0.001**
**Type of previous operations**			
Open only	56 (47.5)	47 (56.6)	**0**.**002**
MIS only	10 (8.5)	7 (8.4)	–
Both	21 (17.8)	24 (28.9)	–
None	31 (26.3)	5 (6.0)	–
**Previous episodes of aSBO**			
0	101 (85.6)	40 (48.2)	**<0.001**
1	9 (7.6)	15 (18.1)	–
2	3 (2.5)	13 (15.7)	–
≥3	5 (4.2)	15 (18.1)	–

Values are *n* (%) unless otherwise indicated. Figures in bold are statistically significant. aSBO, adhesional small bowel obstruction; i.q.r., interquartile range; CCI, Charlson Co-morbidity Index; MIS, minimally invasive surgery.

**Table 2:** Clinicopathological demographics of patients with primary or recurrent adhesional small bowel obstruction on their index admission

	Type of aSBO		*P*
	Primary	Recurrent	
No. of patients	141	60	–
Male:female	0.7	0.9	0.438
Median age (i.q.r.)	70 (56–79)	70 (55–84)	0.709
**ASA grade**			
I	24 (17.0)	7 (11.7)	0.630
II	62 (44.0)	28 (46.7)	–
≥III	55 (39.0)	25 (41.7)	–
**Performance status**			
0	76 (53.9)	26 (43.3)	0.407
1	37 (26.2)	16 (26.7)	–
≥2	28 (19.9)	16 (26.7)	–
Median CCI (i.q.r.)	4.0 (2.5–5.0)	3.5 (1.3–5.0)	0.611
Median previous operations (i.q.r.)	1 (0–2)	3 (2–4)	**<0.001**
**Type of previous operations**			
Open only	68 (48.2)	35 (58.3)	**<0.001**
MIS only	16 (11.3)	1 (1.7)	–
Both	22 (15.6)	23 (38.3)	–
None	35 (24.8)	1 (1.7)	–
**Previous episodes of aSBO**			
1	–	23 (38.3)	–
2	–	16 (26.7)	–
3	–	5 (8.3)	–
≥4	–	15 (25.0)	–
**Treatment of index aSBO**			
Operative	101 (71.6)	17 (28.3)	**<0.001**
Non-operative	40 (28.4)	43 (71.7)	–

Values are *n* (%) unless otherwise indicated. Figures in bold are statistically significant. aSBO, adhesional small bowel obstruction; i.q.r., interquartile range; CCI, Charlson Co-morbidity Index; MIS, minimally invasive surgery.

### Operative management during index admission

Of the 118 patients undergoing operative management during their index admission, 20 (16.9 per cent) had suspected acute bowel ischaemia based on pre-operative CT scanning. A laparoscopic approach was used in 60 patients (50.8 per cent) who were treated operatively during their index admission. Although patients selected for laparoscopy had undergone fewer previous operations (median 1 operation (interquartile range (i.q.r.) 0–2) laparoscopic *versus* 2 operations (i.q.r. 1–2) open, *P* = 0.007), no statistical difference was noted in the proportion of patients who had previously been admitted with SBO (laparoscopic 10.0 per cent *versus* open 19.0 per cent, *P* = 0.197). Conversion to open surgery was necessary in 33 patients (conversion rate 55.0 per cent). The median operative duration was significantly shorter in patients in whom laparoscopy was attempted, regardless of the need for conversion (median 63 min (i.q.r. 35–105) laparoscopic *versus* 103 min (i.q.r. 62–154) open, *P* < 0.001). Iatrogenic injuries were more common in patients undergoing open operations compared with those starting with a laparoscopic approach (44.8 per cent *versus* 18.3 per cent, *P* = 0.002), with double the rate of inadvertent enterotomies (12.2 per cent *versus* 5.0 per cent). However, no difference was noted in the rate of bowel resections (21.4 per cent *versus* 21.7 per cent, *P* = 0.749). Of the 24 patients undergoing bowel resection, iatrogenic injuries were the indication in 9 (37.5 per cent).

### SBO recurrence-free survival

**Fig. 2:**
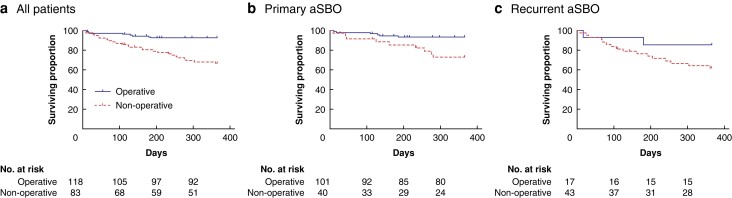
Recurrence-free survival stratified according to management during index admission **a** all patients, **b** patients with primary adhesional small bowel obstruction and **c** patients with recurrent adhesional small bowel obstruction.

A total of 36 patients were readmitted with recurrent SBO within 1 year of their index admission (17.9 per cent). Patients who were treated operatively during their index admission had significantly better recurrence-free survival at 1-year follow-up than those treated non-operatively (operative 92.5 per cent (95 per cent c.i. 85.5–96.2) *versus* non-operative 66.6 per cent (95 per cent c.i. 54.4–75.9), *P* <0.001) (*Fig. 2*). This difference in recurrence-free survival was still noted when only those patients included with their first episode of aSBO were analysed (operative 93.5 per cent (95 per cent c.i. 86.1–97.0) *versus* non-operative 72.9 per cent (95 per cent c.i. 54.2–84.9), *P* = 0.002) (*Fig. 2*). A similar trend was also seen when analysing only those patients included with recurrent aSBO, although this did not reach statistical significance (operative 85.7 per cent (95 per cent c.i. 53.9–96.2) *versus* non-operative 61.7 per cent (95 per cent c.i. 45.2–74.6), *P* = 0.124) (*Fig. 2*).

Of the patients treated operatively, fewer recurrences were seen in patients undergoing a completely laparoscopic operation (0/27 patients) when compared with those undergoing open surgery or in whom conversion was necessary (10/91 patients, *P* = 0.114). However, no difference in the rate of recurrence was seen between patients with band or matted adhesions (band 91.7 per cent (95 per cent c.i. 76.3–97.2) *versus* matted 92.9 per cent (95 per cent c.i. 83.7–97.0), *P* = 0.840). Similarly, recurrence rates did not differ according to the need for bowel resection during the index admission (bowel resection (94.5 per cent (95 per cent c.i. 71.9–99.3)) *versus* none (89.7 per cent (95 per cent c.i. 81.2–94.5)), *P* = 0.402).

### Treatment of recurrence and readmission

**Fig. 3:**
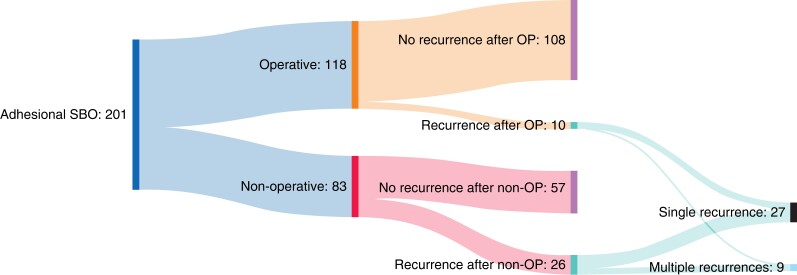
Sankey diagram of recurrences of SBO in patients admitted with adhesional small bowel obstruction OP, operative management on index admission; non-OP, non-operative management on index admission; SBO, small bowel obstruction.

**Fig. 4:**
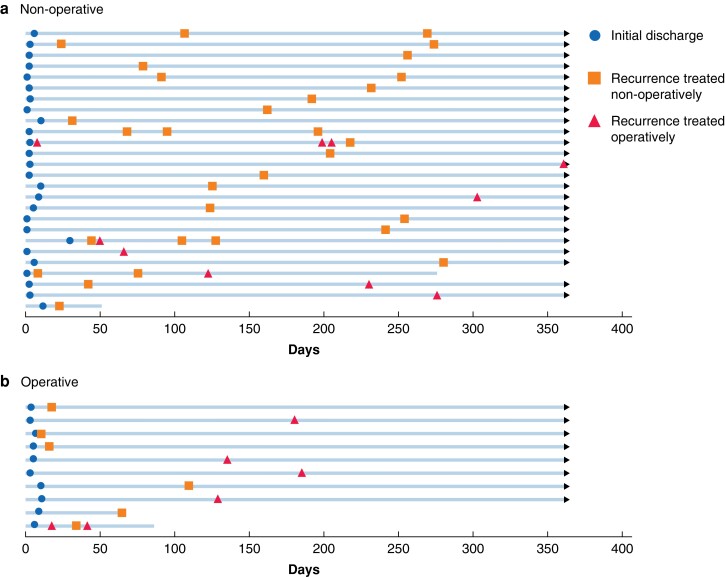
Swimmer plots of recurrences of SBO in patients treated: a non-operatively or b operatively during index admission

*Figures 3* and *4* summarize the treatment of recurrences and patterns of readmissions in patients who developed recurrent SBO after their index admission. Of the 83 patients managed non-operatively during their index admission, 26 (31.3 per cent) developed at least one recurrence of SBO, with eight developing multiple recurrences (9.6 per cent) (*Fig. 4*). Surgical intervention for recurrent SBO was required in eight patients within 1 year of the index admission (9.6 per cent).

Of the 118 patients managed operatively during their index admission, ten developed recurrent SBO (8.5 per cent), of whom only one developed multiple recurrences (0.8 per cent) (*Fig. 4*). Whilst the rate of surgical intervention for recurrences was higher in these patients (5 out of 10 patients), when considering the operative cohort as a whole, surgical intervention for recurrent SBO within 1 year of the index admission was required in 4.2 per cent of patients.

Interestingly, when the length of the index admission was included, patients treated non-operatively spent less time admitted due to SBO during the first year after admission when compared with patients treated operatively, despite their higher rates of recurrence (median 3 days (i.q.r. 2–7) non-operative *versus* 6 days (i.q.r. 4–11.3) operative, *P* <0.001).

### Survival outcomes

A total of 15 patients (7.5 per cent) died within 90 days of their index diagnosis of aSBO. The 90-day mortality rate was higher in the operative (11 patients, 9.3 per cent) compared with non-operative group (4 patients, 4.8 per cent), although this was not statistically significant (*P* = 0.284). At 1-year follow-up from the index diagnosis, a total of 29 patients had died (14.4 per cent). A total of ten deaths were judged to be directly related to aSBO, all of which occurred within 90 days of the index admission.

**Fig. 5:**
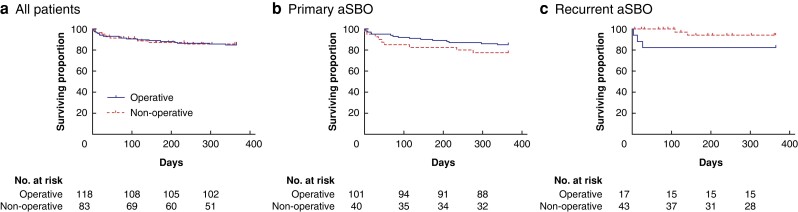
Overall survival stratified according to management during index admission in: a all patients, b patients with primary adhesional small bowel obstruction (aSBO) and c patients with recurrent adhesional small bowel obstruction

No statistical difference in 1-year overall survival was noted between these groups (operative 84.7 per cent (95 per cent c.i. 76.9–90.1) *versus* non-operative 85.6 per cent (95 per cent c.i. 77.3–92.4), *P* = 0.916) (*Fig. 5*). In patients with primary aSBO, 1-year overall survival was slightly higher in those treated operatively than non-operatively, although this was not statistically significant (operative 85.1 per cent (95 per cent c.i. 76.6–90.8) *versus* non-operative 77.5 per cent (95 per cent c.i. 61.2–87.6), *P* = 0.256) (*Fig. 5*). The opposite was seen in patients with recurrent aSBO, with slightly higher overall survival in patients treated non-operatively, although this again was not statistically significant (operative 82.4 per cent (95 per cent c.i. 54.7–93.9) *versus* non-operative 94.2 per cent (95 per cent c.i. 78.7–98.5), *P* = 0.114) (*Fig. 5*).

### Prognostic factors for outcomes

Univariable and multivariable analyses were performed to investigate potential prognostic factors for the recurrence of SBO (*Table 3*) and all-cause mortality rate (*Table 4*). The treatment strategy during the index admission was the only significant prognostic factor for recurrence of SBO, with operative management associated with a decreased risk of recurrence compared with non-operative treatment (HR 0.22 (95 per cent c.i. 0.10–0.48), *P* <0.001). The number of previous operations was not found to be significantly associated with recurrence of SBO. However, operative treatment was also associated with poorer overall survival (HR 2.48 (95 per cent c.i. 1.13–5.46, *P* = 0.024)), alongside poorer performance status and higher CCI.

**Table 3:** Univariable and multivariable Cox regression analyses for recurrence of small bowel obstruction after index admission

	Univariable		Multivariable	
	HR (95% c.i.)	*P*	HR (95% c.i.)	*P*
**Sex**				
Male	Reference	–	Reference	–
Female	1.56 (0.78–3.12)	0.208	1.83 (0.90–3.70)	0.093
Age	1.00 (0.98–1.02)	0.858	*	*
**Performance score**				
0	Reference	–	Reference	–
1	1.70 (0.77–3.75)	0.186	2.23 (1.00–4.98)	0.051
≥2	**2.72** (**1.23–6.01)**	**0**.**013**	1.91 (0.86–4.26)	0.115
CCI	1.11 (0.97–1.28)	0.138	*	*
**Treatment**				
Non-operative	Reference	–	Reference	–
Operative	**0.25** (**0.12–0.51)**	**<0.001**	**0.22 (0.10–0.48)**	**<0.001**
**No. of previous operations**				
0	Reference	–	*	*
1	0.88 (0.25–3.11)	0.838	*	*
2	1.23 (0.35–4.37)	0.745	*	*
≥3	**3.37** (**1.15–9.87)**	**0**.**027**	*	*
**Previous aSBO**				
None	Reference	–	*	*
One	1.63 (0.61–4.40)	0.331	*	*
Multiple	**3.13** (**1.53–6.39)**	**0**.**002**	*	*

Figures in bold are statistically significant. *Excluded from the multivariable model. CCI, Charlson Co-morbidity Index; aSBO, adhesional small bowel obstruction.

**Table 4:** Univariable and multivariable Cox regression analyses for all-cause mortality rate after index admission

	Univariable		Multivariable	
	HR (95% c.i.)	*P*	HR (95% c.i.)	*P*
**Sex**				
Male	Reference	–	*	*
Female	1.68 (0.77–3.69)	0.195	*	*
Age	**1.05 (1.02–1.09)**	**<0.001**	*	*
**Performance score**				
0	Reference	–	Reference	–
1	**7.15 (1.49–34.41)**	**0.014**	3.78 (0.77–18.59)	0.101
≥2	**30.35 (7.08–129.98)**	**<0.001**	**14.81 (3.20–68.64)**	**<0.001**
CCI	**1.56 (1.34–1.82)**	**<0.001**	**1.41 (1.15–1.72)**	**<0.001**
Suspected bowel ischaemia	1.08 (0.33–3.57)	0.900	*	*
**Treatment**				
Non-operative	Reference	–	Reference	–
Operative	1.17 (0.55–2.47)	0.690	**2.48 (1.13–5.46)**	**0.024**
**No. of previous operations**				
0	Reference	–	*	*
1	0.78 (0.27–2.26)	0.649	*	*
2	1.36 (0.49–3.74)	0.553	*	*
≥3	0.50 (0.15–1.64)	0.254	*	*
**Previous aSBO**				
None	Reference	–	*	*
One	0.48 (0.11–2.05)	0.324	*	*
Multiple	0.47 (0.14–1.55)	0.214	*	*

Figures in bold are statistically significant. *Excluded from the multivariable model. CCI, Charlson Co-morbidity Index; aSBO, adhesional small bowel obstruction.

## Discussion

The present study is one of few prospective studies to compare recurrence rates between operative and non-operative management of patients with aSBO and found that operative management is associated with a significant reduction in the risk of subsequent recurrence. Whether presenting with primary or recurrent aSBO, patients treated non-operatively were approximately four times more likely to be readmitted with SBO within the first year of initial admission.

These findings support the results of recent retrospective registry-based studies, that contrary to surgical dogma, have suggested that early operative intervention may be of benefit in patients with aSBO. In a retrospective study of more than 27 000 patients from Canada, operative management was associated with a HR of 0.62 for recurrence compared with non-operative management^11^. In a similar study of over 6000 patients from the USA, operative management was associated with a HR of 0.27 for recurrence^10^. The consistency of these results, from different nations and with different methodologies, is highly suggestive that the benefit of early surgical intervention in reducing the risks of recurrent SBO is genuine.

However, surgical intervention in patients with aSBO is not without risk. Iatrogenic injuries are common in patients undergoing adhesiolysis, with the incidence of inadvertent enterotomies in the present study being consistent with previously reported rates of approximately 10 per cent^14^. It is worth noting that in the present study, over one-third of patients who underwent a bowel resection did so due to an iatrogenic injury. Other adverse effects include chronic abdominal pain and incisional hernias, which have both been reported in approximately 20 per cent of patients undergoing adhesiolysis^15,16^. Operative management has also been found to be associated with increased risk of death. In a previous study, patients undergoing operative management were almost four times more likely to die during initial admission than those managed non-operatively^10^. The severity of clinical presentation is undoubtedly a confounding factor for survival outcomes, with critically unwell patients more likely to be selected for early operative management. In the present study, suspected bowel ischaemia was included in the multivariable analyses to try to account for this. Despite this, operative management was still found to be associated with an increased all-cause mortality rate in the first year after index admission, with a HR of 2.48 when compared with patients treated non-operatively.

The potential benefits of early surgical intervention have led to debate as to whether a more aggressive surgical strategy, which would contradict current international guidelines^17^, is justifiable. It has been argued that whilst a more aggressive strategy may reduce the number of patients developing recurrent SBO, it could paradoxically result in a greater number of patients undergoing, potentially unnecessary, surgery^18^. It has previously been noted that the general approach to patients with SBO is more aggressive in Denmark than in other nations^3^. Indeed, in the present study more than half of all patients with aSBO underwent operative management, and the majority without a previous trial of non-operative management. This rate of operative intervention is roughly twice as high as those reported from other nations^2,8–10^. As such, one may expect the overall recurrence rate for a Danish cohort to be lower than that reported in other studies. It is therefore interesting to note that these recurrence rates are broadly similar, despite the shorter follow-up of the present study, with a recurrence rate of 17.9 per cent at 1 year compared with 19.6 per cent in the study from Canada and 23.8 per cent in the study from USA, both of which have almost 10 years of follow-up^10,11^.

An alternative argument for a more aggressive surgical strategy is that it may be more cost-effective than a non-operative approach. A subsequent cost-benefit analysis based on the same retrospective Canadian cohort found that while total costs were higher for patients undergoing early surgical intervention, this approach was more cost-effective in terms of quality-adjusted life-years^19^. This seems to contradict the findings of the present study, where patients treated non-operatively spent significantly fewer days admitted to hospital when compared with those treated operatively, despite their higher risks of recurrence. Patients successfully managed non-operatively generally have shorter hospital stays, fewer complications and a reduced requirement for intensive care than those undergoing operative management^2,3^.

In reality, the heterogeneity of the patient population presenting with aSBO necessitates an individualized approach to each patient rather than a one-size fits all strategy. The potential benefits of avoiding future recurrences are likely to be far more relevant to a young, fit patient than an elderly patient with multiple co-morbidities, who is at far greater risk from surgical intervention. Similarly, the complexity of surgical intervention differs between a patient who presents with aSBO after a single previous laparoscopic operation and one who has undergone multiple open operations. Whilst the results of the present and previous studies can be used to better inform patients of the potential risks and benefits of different strategies, one factor that is sorely missing from the current literature is the patient’s perspective on the management of this condition. Future studies to determine which outcomes are of most interest to this diverse patient population would be of great interest.

The authors acknowledge the limitations of the present study. The follow-up is short, particularly when compared with other retrospective studies. Recurrence rates for patients treated either operatively or non-operatively are thought to double between 1- and 5-year follow-up^15,20^. As such, it would be of interest to compare outcomes between these groups at a later time point. The cohort size, whilst comparable to other prospective studies of patients with SBO^21,22^, is also small in comparison to the large registry-based studies that have previously been performed on this topic. This is of particular relevance with respect to the multivariable analyses, the results of which, although in keeping with the published literature, should be interpreted with caution.

In conclusion, operative management of aSBO is associated with reduced risks of recurrence within the first year of admission when compared with non-operative management. However, an operative approach is also associated with an increased all-cause mortality rate alongside performance status and CCI. These risks and benefits should be discussed with patients admitted with aSBO when determining the most suitable management strategy, taking into account their co-morbidities and past surgical history.

## Data Availability

In accordance with Danish law the data, on which the findings of this study are based, cannot be made available for sharing.
